# The effect of the online eye movement desensitization and reprocessing early intervention protocol (EMDR R-TEP) for the risk groups with post-traumatic stress symptoms during the COVID-19 pandemic

**DOI:** 10.3389/fpsyg.2022.935782

**Published:** 2022-09-29

**Authors:** Asena Yurtsever, Orkide Bakalim, Şenel Karaman, Sefa Kaya, Emre Konuk

**Affiliations:** ^1^Institute AY, Istanbul, Turkey; ^2^Guidance and Psychological Counseling Department, Educational Sciences Institute, Izmir Democracy University, İzmir, Turkey; ^3^Liman Psychology, Izmir, Turkey; ^4^Psychological Counseling and Guidance Department, Educational Sciences Institute, Pamukkale University, Denizli, Turkey; ^5^Institute for Behavioral Studies, Istanbul, Turkey

**Keywords:** EMDR, early interventions, R-TEP, PTSD, Covid-19, frontline professionals

## Abstract

The aim of the research is to investigate the effect of eye movement desensitization and reprocessing (EMDR) therapy on post-traumatic stress disorder (PTSD) levels of individuals who can be defined as high-risk groups during the pandemic. Therefore, the online EMDR R-TEP Protocol was applied to a total of 154 individuals working with coronavirus patients, frontline professionals (Doctors, Nurses, Paramedics, Polices, Red Crescent), relatives of coronavirus patients, coronavirus patients, and relatives of someone who died from coronavirus and the PTSD symptom level before, after, and 1 month after therapy was measured and examined. A personal information form and impact of events scale were used to collect data. Analyses showed that EMDR therapy was effective in reducing the PTSD level in all groups. The PTSD levels of frontline professionals continued to decrease until the follow-up test but remained the same in the other groups.

## Introduction

A pandemic is defined as a large-scale epidemic disease that can affect many countries or the whole world in a period (World Health Organization, 2010). AIDS, SARS, and H1N1 are among the epidemic diseases that have affected large numbers of people in many countries in the past (Bilge and Bilge, 2020). The recent new type of coronavirus (COVID-19), which appeared in China for the first time in December 2019, has spread around the world since its outbreak, and the World Health Organization (WHO) declared a pandemic on March 11, 2020. According to November 2021 data, during the COVID-19 pandemic, which is still ongoing, the virus has infected approximately 247 million people globally and 5 million cases resulted in death (World Health Organization, 2020). In Turkey, about 8 million people were infected with the virus as of the same date, and about 70,000 people died because of this (Republic of Turkey Ministry of Health, 2020). This situation is a challenge to the psychological health of individuals and societies in addition to their physical health (Holmes et al., 2020). The pandemic’s adverse effects on individuals’ psychological health have been supported by some research findings (Goodwin et al., 2009; Liu et al., 2020; Zandifar and Badrfam, 2020). Pandemics include psychosocial stressors such as the threat to one’s own and loved ones’ health, disruption of routines, separation from loved ones, and social isolation, as well as other stressors such as the inability to bury the deceased naturally (Taylor, 2019). Additionally, the ensuing uncertainty, the necessity of isolation, the risk of transmission of the disease, causing different symptoms in different individuals, and the anxiety of infecting relatives can result in a perception of intense stress as well as feelings such as anxiety and fear (Rubin et al., 2010; Shultz et al., 2015; Van Bortel et al., 2016; Bao et al., 2020; Shevlin et al., 2020; Zhang et al., 2020). Wang et al. (2020) noted in their study with 1,210 participants who were in quarantine during the COVID-19 pandemic that 8.1% of these individuals’ stress levels increased from moderate to high severity within 2 months. According to Huremovic (2019), being sick and seeing loved ones suffer and die, added to the isolation, and when combined with fear, is likely to cause post-traumatic stress disorder (PTSD) in the long run. Especially for individuals whose relatives are hospitalized in intensive care units, not being able to visit them and the fear of not seeing them again are highly stressful experiences (Luttik et al., 2020).

During the COVID-19 pandemic, the group with the highest risk of getting and transmitting the disease has been frontline professionals (Doctors, Nurses, Paramedics, Polices, Red Crescent). In addition to facing risk constantly, the long working hours, excessive fatigue, and lack of systematic training programs on the pandemic due to lack of time, stigma, etc. also affect healthcare professions mental health significantly (Pala and Metintas, 2020; Sakaoğlu et al., 2020). Some recent studies showed that healthcare professions are in the first-degree risk group for having PTSD (Carmassi et al., 2020; Johnson et al., 2020). Feelings of uncertainty and fear of infecting their relatives and coworkers were stated as other contributing variables (Carmassi et al., 2020). Blekas et al. (2020) determined that negative emotions, physical tension, and insomnia were significant predictors of PTSD in healthcare professions.

The risk of PTSD was found to be higher in individuals experiencing more threats and loss of resources during periods such as pandemics (Kaseda and Levine, 2020) This being the case, providing appropriate and effective psychological support to people who develop chronic symptoms such as PTSD in response to traumatic stress is necessary (Rosen et al., 2020). One approach that can provide this psychological support is Eye Movement Desensitization and Reprocessing (EMDR) Therapy, a psychotherapy school based on the Adaptive Information Processing Model (Shapiro, 2017). EMDR therapy has been proven to be effective for PTSD symptoms (Marcus et al., 1997; Van Etten and Taylor, 1998; Maxfield and Hyer, 2002; Konuk et al., 2006; Acarturk et al., 2016; Wilson et al., 2018; Yurtsever et al., 2018), medically unexplained (somatic) symptoms (van Rood and de Roos, 2009), depression, anxiety (Shapiro, 1999; Capezzani et al., 2013), and many other psychological disorders. In fact, in the meta-analysis study by Mavranezouli et al. (2020), it was found that the two most effective interventions for PTSD were Trauma-focused Cognitive Behavioral Therapy and EMDR.

Early EMDR interventions are used to prevent PTSD or ongoing stress in those with traumatic stress symptoms and acute stress disorder, and those at risk for PTSD or other disorders (Shapiro, 2012). Many protocols were developed as the part of early EMDR interventions. Studies were conducted in which these protocols were applied immediately after traumatic social events like natural disasters such as earthquakes and floods (Adúriz et al., 2009; Saltini et al., 2017; Trentini et al., 2018), wars, terrorist attacks, finding evidence of massacres, and military traumas (Zaghrout-Hodali et al., 2008; Wesson and Gould, 2009; Jarero and Uribe, 2011, 2012; Brennstuhl et al., 2019), and the interventions were found to be effective.

In the early EMDR interventions context, the EMDR R-TEP Protocol, which was designed and developed with an inclusive perspective, is an appropriate method for dealing with recent traumas (Shapiro and Laub, 2009). From this point of view, EMDR R-TEP aims to focus on the traumatic episode in recent traumas and to ensure that the whole traumatic process and the memories in this process are perceived and integrated as a consolidated whole and thus adaptively processed. Thus, the traumatic event is called a traumatic episode, which includes the moment that traumatic event has happened till the present moment. All the disturbing pictures, thoughts, feelings, and body sensations are assessed with bilateral stimulation. It aims to help the brain process traumatic events in the acute phase and prevent PTSD in the long term. Various studies have recommended this method to reduce PTSD symptoms after recent trauma (Shapiro and Laub, 2015; Saltini et al., 2017; Shapiro et al., 2018). Accordingly, the circumstances in which EMDR therapy is effective are parallel with the psychological symptoms that arise or may arise during the current COVID-19 pandemic.

The objective of the present study is to investigate the effect of EMDR therapy on PTSD levels of individuals who could be defined as high-risk groups during the pandemic. Therefore, the online EMDR R-TEP Protocol was applied to individuals working with coronavirus patients, frontline professionals, relatives of coronavirus patients, coronavirus patients, and relatives of someone who died from coronavirus, and the PTSD symptom level before, after, and 1 month after therapy was measured and examined.

## Materials and methods

### Research model

The aim of this study was to examine the effect of five sessions of EMDR R-TEP intervention on the PTSD levels of individuals affected by the COVID-19 pandemic. A single group pre-test-post-test-follow-up (1 × 3) design was used. In this design, the first factor shows the independent process groups (experiment) and the second factor shows the repeated measurements of the dependent variable (pre-test, post-test, and follow-up) under different conditions (Büyüköztürk et al., 2013). The study’s independent variable is the EMDR R-TEP intervention and the dependent variable is the PTSD levels of individuals affected by the coronavirus.

### Participants

Coronavirus patients, relatives of coronavirus patients, relatives of someone who died from coronavirus, professions working with coronavirus patients, and frontline professionals, who can be defined as high-risk groups during the coronavirus pandemic, participated in the study. The EMDR R-TEP intervention was applied to 745 people who met these criteria. Only 154 of them completed the pre-test, post-test, and follow-up test and included as the participants of this research. They were reminded to fill in, but due to voluntariness, late participants who filled in whenever they wanted could not be included. In addition, the forms of the clients who could not use the internet well, although they filled it in at the right time, the forms reached us late and could not be included.

### Process

**Figure 1:**
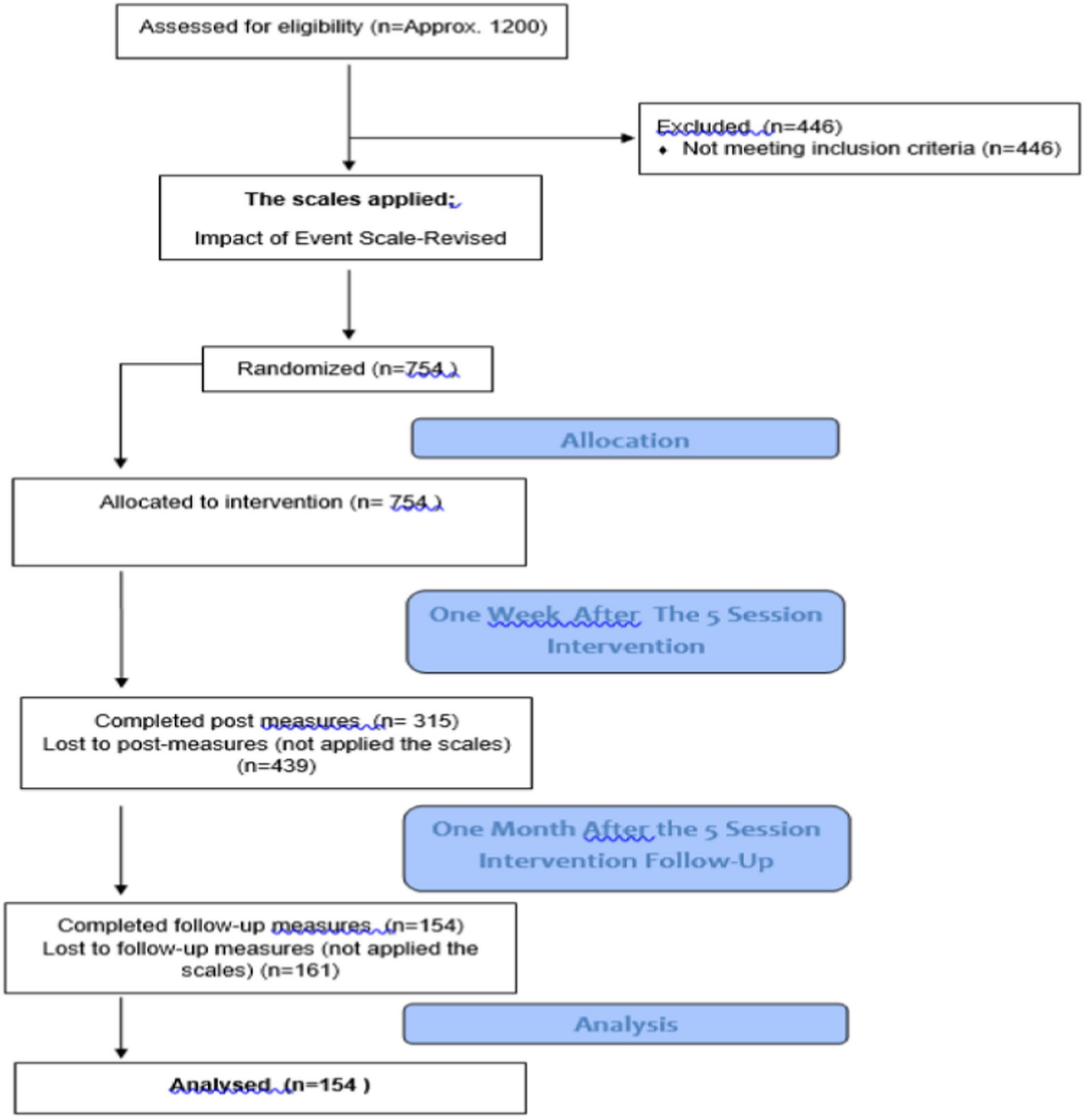
Flowchart of participants through each stage of the study.

Before the research started, research permission was obtained from the Social and Human Sciences Scientific Research and Publication Ethics Committee of Izmir Democracy University (Protocol No: 2020/50; Acceptance Date: 07.08.2020; Decision Number and No: 2020/10–06). In order to carry out the trauma recovery group coronavirus study, a unique organization was made specifically for the coronavirus study. Therapists who had at least first-level EMDR Europe accredited EMDR training and voluntarily applied to the trauma recovery group were included in this study. These therapists were given online EMDR training and EMDR R-TEP training. 450 EMDR therapist applied and they were divided into two groups that will perform EMDR therapy and as support teams. The groups that will do EMDR therapy are divided into sub-teams of 20 people. At the head of each team, one person (team leader) provides communication with the main organization and also leads his own team. Support teams are; social media team, WhatsApp team, mail team, project team, morale team (the team that provides psychological support to the volunteer therapists who were psychologically affected for any reason during the project process) and the management coordination team. After establishing the teams, the project was announced to the public *via* social media accounts for five and a half months, from March 16, 2020, when the study on the coronavirus epidemic began, until September 01, 2020, when it was completed, and the project received coverage in the visual and written media. Those who were aware of this project were asked to send a message to the relevant WhatsApp number or email account in order to apply the project. Then a standard consent form and client information form were sent to the applicants. After filling out this form, our inclusion criteria were belonging to one of the four groups we studied and not having a psychiatric diagnosis. If this was not specified on the form, we informed those candidates directly that we would not be able to give service The clients who are matching the criteria were directed to the most appropriate team leader by the team member in the management. The team leader primarily directs the referred client to the most experienced EMDR therapist in his team. Thus, less experienced therapists have the chance to follow the supervision of previous cases. While assigning the client, the information including the personal information form, consent form, and impact of events scale were sent to the therapist. The therapist made an appointment by calling the assigned client. The therapist was asked to plan the therapy for five sessions, in accordance with the EMDR Trauma Recovery Group rules and to get supervision in this regard. The therapist sent the Impact of Events Scale to the client 1 week and 1 month after the five sessions intervention were completed. In the first session the therapist checks if the clients need any psychiatric support and they referred the client to the Psychiatric Advisory Board within the EMDR Trauma Recovery Group. S/he also recorded the entire therapy process on the Therapist Information Entry form. Finally, each client had the five session of EMDR therapy in this project. Below the flowchart of participants through each stage of the study is shown in Figure 1.

### Training and supervision of therapists

Between March 2020 and September 2020, 450 volunteer EMDR therapists conducted online EMDR and psychological support work for those affected by COVID-19 pandemic and those who applied upon the invitation from the Turkey EMDR Trauma Recovery Group (Karaman et al., 2020). Only persons who have received accredited EMDR therapy training and are registered with the EMDR Association can participate in EMDR Trauma Recovery Group studies and projects. After the volunteer therapists were accepted for the project, they had in-service training related to the field they would work in, Information on the training and supervision periods of the therapists is given in Table 1.

**Table 1:** Baseline characteristics of participants.

**Variables**	**Total *N*: 154**
Age, mean	36,32
**Sex, *n* (%)**	
Male	23(%14,9)
Female	131(%85,1)
**Education, *n* (%)**	
Primary school graduates	8
Secondary school graduates	6
High school graduates	11
University graduates	65
Higher education	29
Blank	35

## Data collection tools

### Personal information form

This is the form the researchers created to determine the sex, age, education level, and reason for applying for therapy of the participants in the study group.

### Impact of events scale (IES-R)

Weiss and Marmar (1997) developed the impact of events scale to evaluate the severity of PTSD symptoms in individuals. The scale, which is a self-reported measurement tool, consists of 22 items. It is a five-point Likert-type scale (0 = never, 4 = extreme). Since each item varies between 0 and 4, the highest score that can be obtained from the scale is 88 and the lowest score is 6. A high score on the scale indicates a high severity of PTSD symptoms. The scale consists of three sub-dimensions. The subscales comprise re-experiencing, avoidance, and hyperarousal. The test–retest correlation coefficients of the original scale ranges from 0.57 to 0.94 for intrusion, from 0.51 to 0.89 for avoidance, and from 0.59 to 0.92 for hyperarousal. The scale was adapted into Turkish by Çorapçıoğlu et al. (2006). The IES-R has high internal consistency and the Cronbach’s alpha internal consistency coefficient of the scale was calculated as 0.94. In the present study, the Cronbach’s alpha internal consistency coefficient of the scale was 0.84 and all analyses were conducted on the total score.

### Intervention

The EMDR R-TEP Protocol, which was designed and developed in the context of early EMDR interventions, was carried out online in this process. The EMDR R-TEP covers all eight phases of EMDR therapy as a method used for recent traumas: History, preparation, assessment, desensitization, installation, body scan, closure, and reassessment. It involves stabilization, containment, and creating a sense of security since the traumatic experience has happened relatively recently or is still ongoing. Due to the nature of recent trauma, it is recommended to apply it on consecutive days. Within the scope of the present research, five sessions are enough, only an additional session if necessary were arranged with clients. A pre-test was conducted before the therapy process, a post-test 1 week after the therapy process ended, and a follow-up measurement 1 month after the therapy process ended.

### Fidelity

During the process, in-service training was given to the volunteer therapists. In addition, all therapists participated in group supervision once a week during the process. In the project, 17 supervisors who supervised the groups attended to the supervisors’ supervision sessions once a week.

### Data analysis

Descriptive statistics were calculated for the qualifications of the participants. The Kolmogorov–Smirnov test was used to determine whether the data were normally distributed to decide whether to use parametric or non-parametric analyses in the research. In the Kolmogorov–Smirnov tests for the normality of the distribution of the pre-, post-, and follow-up test scores for the four groups, the *p*-values were completely not significant. For this reason, it was accepted that the distributions were normal, and it was decided to use parametric statistics within the scope of the research. Repeated measures one-way ANOVA was performed to determine the difference in scores between the groups’ pre-, post-, and follow-up test IES-R scores. Before the ANOVA, the sphericity test was performed for the total and all subgroups, and the assumption was met. The Bonferroni test, a *post-hoc* technique, was used to determine from which pairs the determined differences originated. All analyses within the scope of the research were carried out using SPSS 22 (IBM Corp., 2015).

## Results

**Figure 2:**
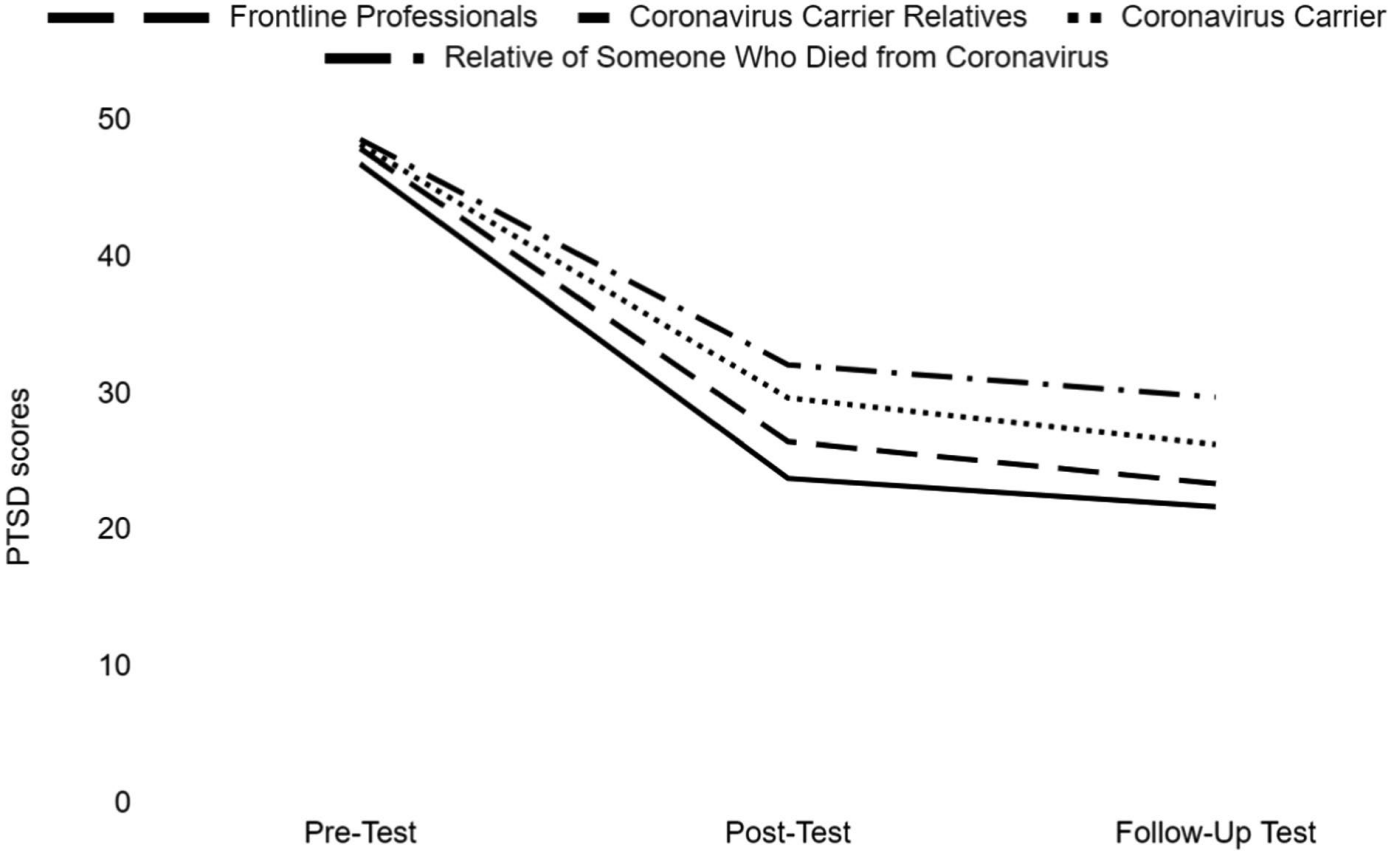
Descriptive graph of the study groups.

The mean and standard deviations of the pre-test, post-test, and follow-up test PTSD scores of the study groups within the scope of the research are shown in Table 2, and a graph showing the pre-test, post-test, and follow-up scores of the study groups is given in Figure 2.

**Table 2:** Therapist support meetings and durations.

Supervision sessions	410 Sessions (each session is 1–3 h)
Supervisors meetings	20 meetings
Support team meetings	220 h
Therapy team meetings	660 h
Number of in-service training sessions	10 sessions
Instagram live broadcasts	9 broadcasts

There was a significant difference between the pre-test, post-test, and follow-up test mean scores of frontline professionals group (*F* (1.697, 140,815) = 168,658, *p* < 0.001), relatives of coronavirus patients group (*F* (1.485, 35,644) = 38,211, *p* < 0.001), coronavirus patients group (*F* (1,263, 26.517) = 32,479, *p* < 0.001) and relatives of someone who died from coronavirus group (*F* (12;44) = 24.539, *p* < 0.001). Also there is a statistically significant difference at the level of 0.05 between the post-test and the follow-up test of frontline professionals. On the other hand, there was no statistically significant difference between the post-test and the follow-up test of the relatives of coronavirus patients, coronavirus patients and relatives of someone who died from coronavirus (Table 3).

**Table 3:** Descriptive statistics of the study groups.

Study group	*N*	Pre-test		Post-test		Follow-test		Between time effect size
		Mean	Std. deviation	Mean	Std. deviation	Mean	Std. deviation	
Frontline professionals	84	47.92	11.72	26.48	11.97	23.41	13.51	0.67^**^
Coronavirus Patients Relatives	25	46.80	11.32	23.79	11.82	21.72	10.19	0.61^**^
Coronavirus Patients	22	48.22	13.10	29.68	16.03	26.27	17.09	0.60^**^
Relatives of Someone Who Died from Coronavirus	23	48.60	13.31	32.11	16.62	29.73	14.77	0.52^**^

** *p* < 0.01.

## Discussion

The present research was conducted with health and similar professions, relatives of coronavirus patients, coronavirus patients, and relatives of someone who died from coronavirus, which can be defined as high-risk groups during the COVID-19 pandemic. Within the scope of the research, the effect of the 5-session online EMDR R-TEP protocol on PTSD levels was examined.

The analysis of the research findings by the groups determined that there was a significant decrease in the pre-test, re-test, and follow-up test PTSD scores of the frontline professionals who underwent EMDR R-TEP. Considering that due to COVID-19 pandemic frontline professionals working without taking breaks, have to work under intense stress, and have a higher number of patients than usual; the traumatic symptoms decreased rapidly and continued to decrease after the sessions indicates the effectiveness of the practice. This finding is consistent with the results reported by Perez et al. (2020) on frontline professionals during the COVID-19 pandemic. In their study, the EMDR-IGTP group treatment protocol, which is another protocol of EMDR therapy with ongoing traumatic stress, was applied. The research was conducted remotely and it revealed that the PTSD, anxiety, and depression results of frontline professionals working with coronavirus patients decreased significantly after the application. Similarly, Moench and Billsten’s (2021) research findings showed that EMDR therapy applied to mental health professions during the COVID-19 pandemic was effective in reducing depression, anxiety, and stress levels. In addition, it has been demonstrated that EMDR therapy applied to emergency nurses during the COVID-19 pandemic provides a decrease in COVID-19-related anxiety and depression levels and an increase in sleep quality (El-Abbassy et al., 2021).

Although there was no statistically significant difference between the post-test and the follow-up test of the relatives of coronavirus patients, another subgroup of the study, the IES-R score averages decreased significantly from the pre-test to the post-test. The EMDR R-TEP practice significantly reduced the symptoms of traumatic stress, only in the group the change did not increase in the follow-up period but continued to be the same that can be seen as the effect remain the same. There was no research finding either supporting or not supporting this result. It is thought that different variables that we could not monitor and control, as we were unable to access information about the progression of the diseases of the relatives after the intervention, may have been reflected in the findings.

It is observed that traumatic symptoms decreased after EMDR R-TEP application in the coronavirus patients group and continued decreasing 1 month after the study. But there was no statistically significant difference between the post-test and follow-up test of the coronavirus patients group. This result shows the positive effect of the implication remained the same after 1 month. In the study conducted by Jelveh (2021), EMDR therapy was applied to 19 patients hospitalized due to COVID-19 pandemic, and a significant decrease in the anxiety levels of the patients after the therapy was determined. However, more research is needed to reveal the effect of EMDR therapy on PTSD during the COVID-19 pandemic (Lenferink et al., 2020).

A significant decrease in pre-test–post-test and post-test–follow-up test scores was observed in the group who lost relatives due to coronavirus. However, there was no change in the post-test and follow-up test. In other words, the intervention reduced the PTSD levels of the individuals, and this continued during the monitoring process. This group is in mourning because of the loss they experienced, and still the decrease in traumatic symptoms similar to other groups. Considering the hypothesis (Solomon, 2018) that for a successful grieving process, traumatic material that may interfere with this process is processed by the brain using the EMDR protocol, it can be inferred that the practice might support a healthy grieving process. The research conducted by Solomon and Hensley (2020) presented data indicating that EMDR therapy can be effective in individuals who have lost relatives due to coronavirus. In fact, some previous research findings demonstrated that EMDR plays a supportive role in a healthy grieving process (Sprang, 2001; Hornsveld et al., 2010; Usta et al., 2016; Solomon, 2018). Examination of the PTSD levels of the total study group showed that EMDR therapy effectively reduced PTSD symptoms in the whole group, and this effect continued with a significant decrease after 1 month. Similar to this finding, studies have revealed that processing memories in EMDR therapy continue after the sessions (Shapiro, 2017). Perri et al. (2021) conducted research comparing the effectiveness of online EMDR R-TEP and online cognitive behavioral therapy for trauma associated with quarantine, isolation, and working in a hospital environment during the COVID-19 pandemic. The findings showed that both therapy methods were effective in reducing PTSD symptoms. On the other hand, it has been found in different studies that the number of similar sessions in different traumatic experiences effectively reduces the level of PTSD. In the study conducted by Saltini et al. (2017), 2–4 sessions of EMDR R-TEP were applied to individuals affected by trauma after an earthquake. It was determined that there was a significant decrease in the stress level of the participants after EMDR R-TEP.

### Strengths and limitations

It is thought that the supervision supporting the therapists during the process of helping these groups increases the power of the research design. However, the research has some limitations. The first of these is that our study has no control group. There is a number of arguments on natural regress of post traumatic symptoms during the first 3 months. Riggs et al. (1995) examined 84 non-sexual assault victims after the assault and continuing weekly for 3 months. Although 71% of the women and 50% of the men met symptom criteria for PTSD at the first assessment, 21% of the women but none of the men remained with PTSD at the final assessment. But in contrary, some who were not diagnosed with PTSD at the final assessment retained significant symptoms of PTSD, particularly re-experiencing and arousal symptoms. In order to eliminate this factor studies with true experimental research design or quasi-experimental research design studies needs to be done. Covers et al. (2021) found out that there is no difference in the watchful waiting group who provided psychoeducation and emotional support and two sessions of EMDR therapy group in reducing post traumatic symptoms, depression, sexual dysfunction, feelings of guilt and shame in the rape victims. Our findings should be supported more by randomized controlled trials with control groups. Consequently, our study was a project in which approximately 1,200 applications were made in 7 months, who wanted to participate and get treatment voluntarily from all over Turkey in the first wave of the Covid-19 pandemic. Therefore, it was not possible to create a control group during this period when early intervention was required. Secondly, although the EMDR-R-TEP intervention was applied to 745 people who were in the risk group throughout the process, pre-test, post-test, and follow-up test results were obtained from only 154 of them. The participants with incomplete forms not included in the study resulted in data loss. Filling out the forms online caused problems either not filled out or not filled in at the specific time. We recommend to future studies to using a preventable online system for this problem. We also suggest conducting a similar study with a control group; conducting studies on the effects of online EMDR therapy on different variables such as anxiety, depression, and intolerance to uncertainty during the pandemic; and conducting new studies comparing online EMDR with different therapy methods during the pandemic.

## Conclusion

In conclusion, the present research showed that online EMDR therapy significantly reduced the PTSD level of individuals in some risk groups during the COVID-19 pandemic. EMDR R-TEP is a recent event protocol. It was used for ongoing trauma in this study. Based on the results, it can be said that EMDR R-TEP can also be used in ongoing traumas.

## Data availability statement

The raw data supporting the conclusions of this article will be made available by the authors, without undue reservation.

## Ethics statement

The studies involving human participants were reviewed and approved by Social and Human Sciences Scientific Research and Publication Ethics Committee of Izmir Democracy University (Protocol No: 2020/50; Acceptance Date: 07.08.2020; Decision Number and No: 2020/10-06). The patients/participants provided their written informed consent to participate in this study.

## Author contributions

AY, OB, ŞK, SK, and EK contributed to conception and design of the study. AY, OB, and SK wrote sections of the manuscript. AY, ŞK, SK, and EK organized the database. AY and SK performed the statistical analysis and wrote the first draft of the manuscript. All authors contributed to the article and approved the submitted version.

## Conflict of interest

The authors declare that the research was conducted in the absence of any commercial or financial relationships that could be construed as a potential conflict of interest.

## Publisher’s note

All claims expressed in this article are solely those of the authors and do not necessarily represent those of their affiliated organizations, or those of the publisher, the editors and the reviewers. Any product that may be evaluated in this article, or claim that may be made by its manufacturer, is not guaranteed or endorsed by the publisher.
